# The larva of *Drusus dudor* Oláh, 2017, including an updated key to larval Drusinae Banks, 1916 (Insecta, Trichoptera, Limnephilidae)

**DOI:** 10.3897/zookeys.908.47032

**Published:** 2020-02-03

**Authors:** Simon Vitecek, Jan Martini, Carina Zittra, Hendrik Kuhlmann, Ariane Vieira, Johann Waringer

**Affiliations:** 1 WasserCluster Lunz, Dr. Carl Kupelwieser Promenade 5, A-3293 Lunz am See, Austria WasserCluster Lunz Biological Station Lunz Austria; 2 Department of Limnology and Bio-Oceanography, University of Vienna, Althanstrasse 14, A-1090 Vienna, Austria University of Vienna Vienna Austria; 3 Institute of Fluid Mechanics and Heat Transfer, TU Wien, Tower BA/E322, Getreidemarkt 9, A-1060 Vienna, Austria Technical University Vienna Vienna Austria

**Keywords:** description, distribution, identification, larval taxonomy, morphology, Palearctic fauna

## Abstract

The caddisfly *Drusus dudor* Oláh, 2017 (Limephilidae: Drusinae) was described from the Northwestern Italian Alps. We provide a detailed description of the larva, based on material from the Italian Province of Piemonte. Information on the morphology of the 5^th^ larval instar is given, and the most important diagnostic features are illustrated. The larva is included in an updated key to larval Drusinae where *D. dudor* keys together with *Drusus aprutiensis* Moretti, 1981, *D. camerinus* Moretti, 1981, *D. croaticus* Marinkovic-Gospodnetic, 1971, *D. mixtus* (Pictet, 1834), and *D. nigrescens* Meyer-Duer, 1875. The species can be reliably separated by the morphology of the pronotum, the shape of the metanotal sclerites, and by morphological details of abdominal sternum I.

## Introduction

The limnephilid subfamily Drusinae is characterized by larvae restricted to high-gradient, turbulent, running waters in hard-substrate channels. Drusinae species occur across the Eurasian mountain ranges from the Iberian Peninsula to the Iranian Highlands. In their larval stages, Drusinae have evolved an amazing diversity of body morphologies coupled with unique feeding strategies; the mechanisms behind these diversifications are not clear, but they are likely due to evolutionary opportunities formed by variable and limited food availability in space and time, the ever-changing hydraulic stress of their lotic environment, or the presence of predators (Pauls et al. 2008). Omnivorous shredders (e.g., *Drusus alpinus* Meyer-Duer, 1875) reflect a potentially basic evolutionary level within the subfamily as they share many characters with their limnephilid congeners in other subfamilies. This entails teeth along the edges of the mandibles without further specializations of the body and characterizes an omnivorous, shredder feeding type (Graf et al. 2009). Species of this group prefer springs and spring brooks. Species in the second group that comprises epilithic grazers only, develop mandibles without terminal teeth as an adaptation to their feeding ecology (e.g., *Drusus nigrescens* Meyer-Duer, 1875; *Drusus bolivari* (McLachlan, 1880); Vieira-Lanero et al. 2005). Finally, larvae of filtering carnivores exhibit toothed, shredder mandibles, filtering spines on legs and the first abdominal sternum, and unique head capsule complexities, unknown in other caddisfly larvae. In this last group, species with bare, flattened, or concave heads are basal to a strongly supported clade comprising all other carnivorous Drusinae (Vitecek et al. 2015). These larvae develop a modified setation or flocculent hair cover on their strongly modified head capsule (e.g., *Drusus discolor* (Rambur, 1842)). In addition, all filtering carnivores develop four distinct cephalic setae that likely serve as sensory organs to detect the optimal hydraulic niche for filtering.

About 75% of the known Drusinae species are endemics limited to a single or very few mountain ranges, and potentially undergo accelerated evolutionary processes including speciation and diversification; this is reflected by the high species diversity of this group: in his ‘Trichoptera World Checklist’, Morse (2019) lists a total of 174 extant Drusinae species with only a fraction of them known in the larval stage. In the present paper we address this lack in larval taxonomy by describing the larva of *Drusus dudor* and by including this larva in a comprehensive larval key to Drusinae.

## Materials and methods

Larvae were collected from the mineral substrate with forceps, and adults were collected using a sweep net. The material was preserved in 70% ethanol. The larvae were studied and photographed using a Nikon SMZ 1500 binocular microscope with DS-Fi1 camera and NIS-elements D 3.1 image stacking software for combining 9–66 frames in one focused image. Larval morphological features follow the nomenclature proposed by Wiggins (1998) and Waringer and Graf (2011), while nomenclature of primary setae and setal areas (= sa) follows Wiggins (1998). Species association was enabled by the fact that larvae and adults were collected at the same site where potentially confusing species were lacking and larvae of all other Drusinae species recorded from the area were known. In addition, collecting sites were close to the type locality of the holotype of *Drusus dudor*. Final instar larvae and adults used for the descriptions are deposited in the collection of S. Vitecek and J. Waringer (Lunz and Vienna, Austria). Comparative larval material of *Drusus chauvinianus* (Stein, 1874) (= *Anomalopterygella chauviniana* (Stein, 1874), *Drusus alpinus* Meyer-Duer, 1875, *D. aprutiensis* Moretti, 1981, *D. bolivari* (McLachlan, 1880), *D. botosaneanui* Kumanski, 1968, *D. camerinus* Moretti, 1981, *D. carpathicus* Dziedzielewicz, 1911, *D. croaticus* Marinkovic-Gospodnetic, 1971, *D. franzi* Schmid, 1955, *D. franzressli* Malicky, 1974, *D. macedonicus* Schmid, 1956, *D. melanchaetes* McLachlan, 1876, *D. mixtus* (Pictet, 1834), *D. monticola* McLachlan, 1876, *D. muelleri* McLachlan, 1868, *D. nigrescens* Meyer-Duer, 1875, *D. ramae* Marinkovic-Gospodnetic, 1971, *D. serbicus* Marinkovic-Gospodnetic, 1971, *D. vinconi* Sipahiler, 1992, *Ecclisopteryx asterix* Malicky, 1979 and *E. dalecarlica* Kolenati, 1848 is deposited in the collection of J. Waringer (Vienna, Austria). The larval material is intended to be subsequently transferred to Austrian Museum collections.

## Results

### Description of the 5 ^th^ instar larva of Drusus dudor

Taxon classificationAnimaliaTrichopteraLimnephilidae

0A9737AE-344B-548B-A79B-EDDC386CF989

#### Biometry.

Body length 10.3–13.8 mm, head width 1.40–1.60 mm (*N* = 6).

#### Head.

**Figures 1–3.**
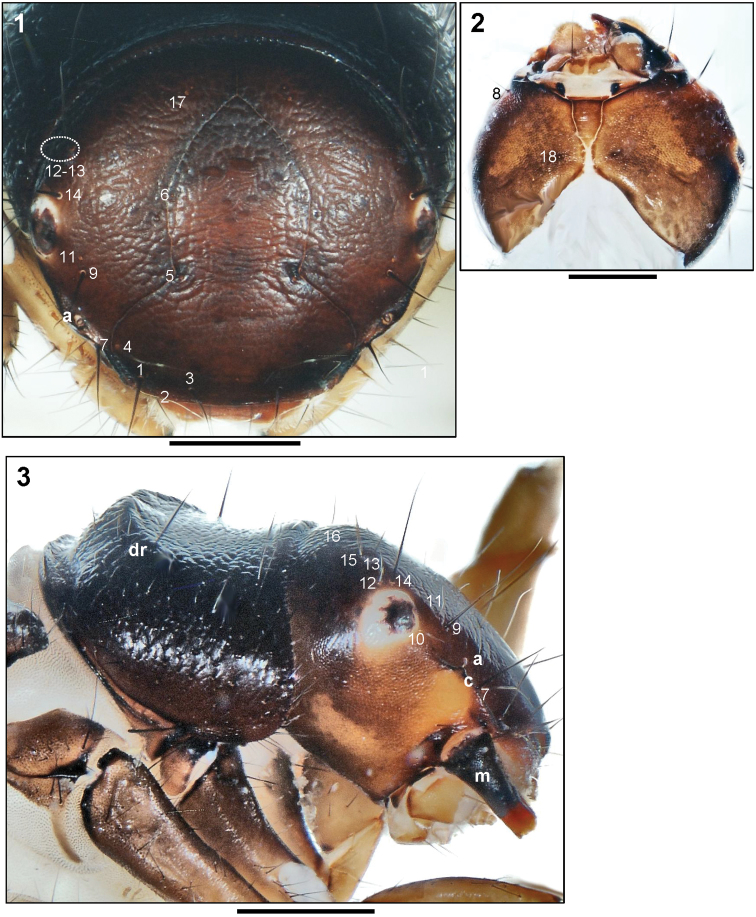
*Drusus dudor* Oláh, 2017, 5^th^ instar larva. **1** Head, dorsal view (a: antenna; numbers refer to setal positions) **2** Head, ventral view (numbers refer to setal positions) **3** Head and prothorax, right lateral view (a: antenna; c: lateral carina; dr: dorsal ridge; m: mandible; numbers refer to setal positions). Scale bars: 0.5 mm.

Head capsule coarsely granulated, outline circular, hypognathous (Figs 1, 3), dorsally chestnut to black brown, with blackish muscle attachment spots. Ventral parietal sections, ventral apotome, maxillolabial sclerites, and premandibular areas yellowish (Figs 2, 3). Eyes surrounded by whitish ring (Fig. 3). In lateral view, head capsule with longitudinal carina extending from anterior border of whitish eye ring to anterior parietal border (Fig. 3c) with antennae located dorsally on central section of carina (Fig. 3a). Antenna consisting of 1 short cylindrical base and 1 prominent lateral seta. Frontoclypeal apotome bell-shaped, with narrow median constriction (Fig. 1). Head capsule with complete set of 18 pairs of primary setae (sensu Wiggins 1998), without additional spines or spinule areas as known from Drusinae grazer clade Groups A and B (Key 1 of the present paper). Primary set of parietal setae consisting of 10 dorsal and 2 ventral primary setae (some of them numbered in Figs 1–3), frontoclypeal apotome bearing 6 pairs of primary setae, 3 of them along anterior border. Labrum yellowish brown, anterolateral margins with setal brush and primary setae 1–3; dorsally, setation consisting of primary setae 4–6 (not visible in Fig. 1). Ventral apotome cuneiform, medium brown anteriorly, yellowish brown posteriorly, with dark brown anterior transverse suture, postgenal suture reaching approximately 35% of apotome length (Fig. 2). Mandibles black brown (sometimes brownish on distal half; Fig. 3), spoon-shaped, lacking terminal teeth along edges as well as ridges in central concavity (Fig. 3).

#### Thorax.

**Figures 4–11.**
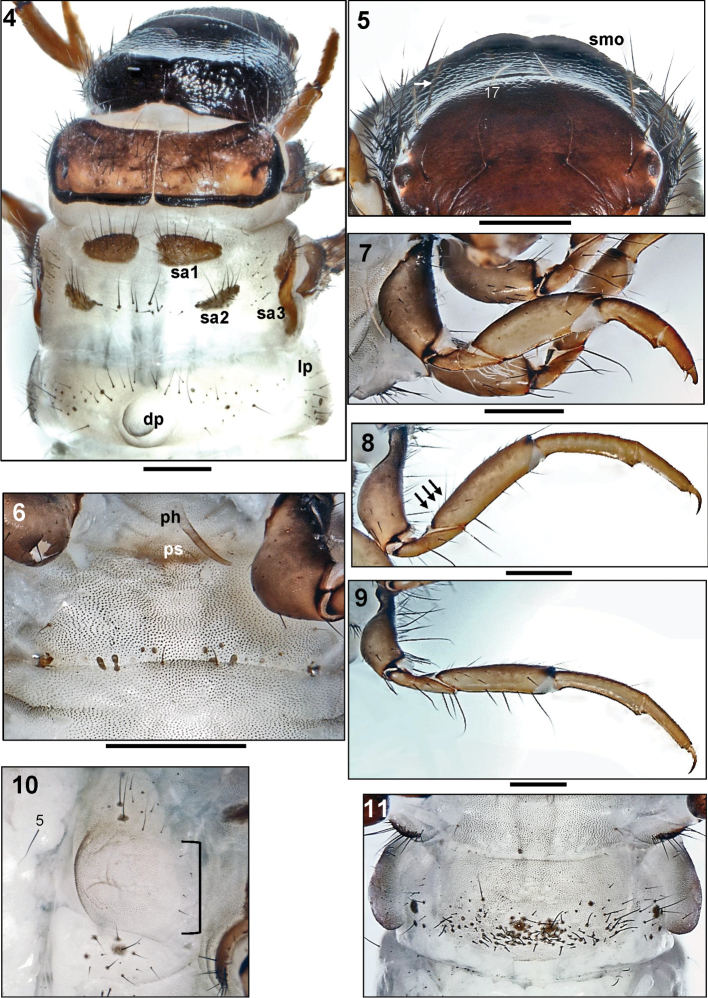
*Drusus dudor* Oláh, 2017, 5^th^ instar larva. **4** Head, thorax and abdominal segment I, dorsal view (dp: dorsal protuberance; lp: lateral protuberance; sa1, sa2, sa3: metanotal setal areas 1, 2 and 3) **5** Head and pronotum, frontal view (arrows: long dark setae not reaching dorsal midline; smo: smooth outline of pronotal ridge; numbers refer to setal positions) **6** Prosternum, ventral view (ph: prosternal horn; ps: prosternite) **7** Left fore leg, anterior view **8** Left mid leg, anterior view (arrows: proximodorsal setae) **9** Left hind leg, anterior view **10** Lateral protuberance, right lateral view (bracket: anterolateral setae; number 5 refers to setal position) **11** Abdominal sternum I, ventral view. Scale bars: 0.5 mm.

Pronotum chestnut brown, very coarsely granulated (Figs 3–5), posterior margin thickened, fitted with black stripes; no pronotal transverse groove at end of anterior third (Fig. 3). In lateral view, with distinct transverse ridge in its posterior third (Figs 3, 4) extending laterally, thereby decreasing in height until ridge is completely faded within lateral center of pronotum (Fig. 3, dr). In anterior view, ridge with smooth outline (Fig. 5, smo) and with shallow, V-shaped central notch (Fig. 5). Along anterior pronotal border 2 setal rows present, including: i) dense fringe of short, curved, fine, yellow setae, ii) row of widely-spaced long, straight, dark setae not reaching pronotal midline (Fig. 5, arrows) [in 1 out of 6 larvae, long, straight, dark setae also present near pronotal midline]. Each pronotal half bearing in total 55–63 dark setae of varying lengths in addition to tiny, pale, curved, recumbent setae in low numbers; without spines present in other Drusinae (e.g., *D. trifidus*). Prosternite inconspicuous, pentangular, pale yellow, with brown posterior border (Fig. 6, ps); prosternal horn present (Fig. 6, ph). Mesonotal sclerites yellowish brown, with dark brown muscle attachment spots and black lateral and posterior borders (Fig. 4). Counts for mesonotal setae in anterior setal group sa1: 8–11, in posterior group sa2: 17–20 and in lateral group sa3: 35–46 (Fig. 4). In addition, small number of tiny, pale, curved, recumbent setae present. Anterior metanotal sclerites (sclerites of sa1, sensu Wiggins 1998) dark brown, large, broadly triangular, their median separation smaller than their length along the longitudinal body axis (Fig. 4, sa1); with 7–21 setae. Posteromedian sclerites (sclerites of sa2) dark brown, triangular, with 12–15 setae per sclerite; with 10–12 setae between them (Fig. 4, sa2). Lateral sclerites (sclerites of sa3) medium to yellowish brown, with 33–37 setae concentrated in cranial section (Fig. 4, sa3). Groups of 7–9 setae present between sa2 and sa3 (Fig. 4). Legs yellowish brown with numerous setae on coxae, trochanters, and femora; tibiae and tarsi sparsely setose (Figs 7–9). Foreleg coxa, femur, and tibia wider than those of mid- and hind legs (Fig. 8). Femora with several proximodorsal setae (e.g., Fig. 8, black arrows), and with additional setae on anterior and posterior faces. Fore femora with 4 pale ventral-edge setae, mid and hind femora each with 4 dark ventral-edge setae. Fore trochanters with distal ventral trochanteral brush. Mid- and hind tibiae with dorsal setae only on distal third (Figs 8, 9). Tarsal claws sickle-shaped, pointed, with basal spur (Figs 7–9).

#### Abdomen.

**Figures 12–17.**
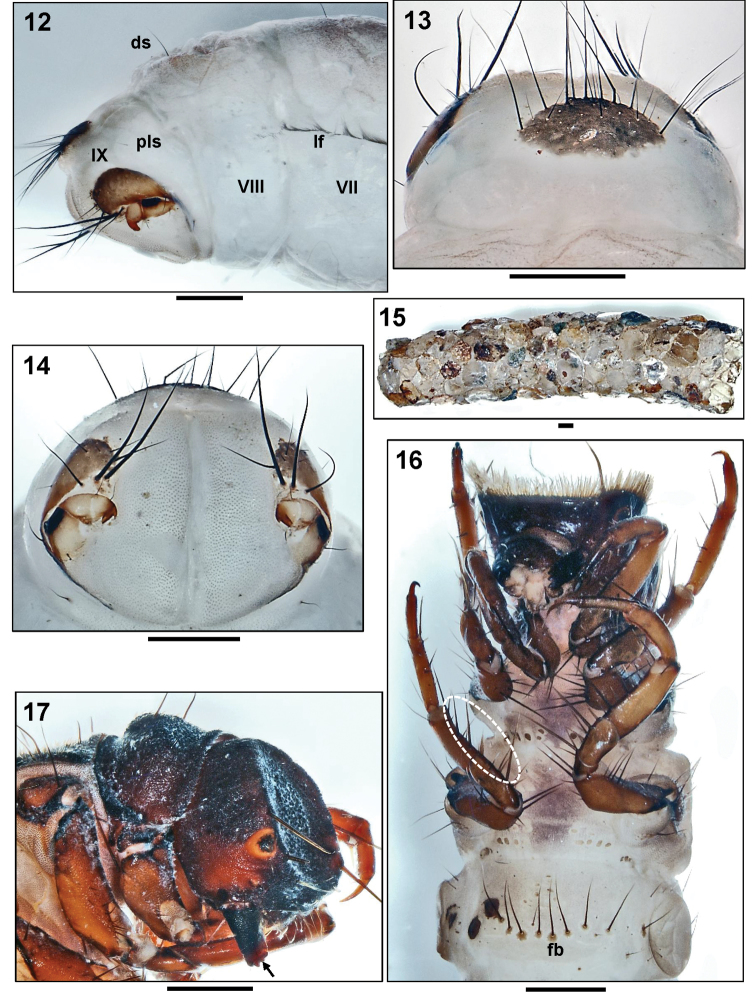
**12–15.***Drusus dudor* Oláh, 2017, 5^th^ instar larva. **12** Abdominal segments VII–IX, right lateral view (lf: lateral fringe; pls: posterolateral seta; ds: dorsal seta) **13** Abdominal segment IX, dorsal view **14** Tip of abdomen, posterior view **15** Larval case, right lateral view **16***Drusus macedonicus* Schmid, 1956, 5^th^ instar larva, head, thorax and abdominal segment I, ventral view (fb: filtering bristles, dotted oval: filtering bristles on hind femur) **17***Drusus muelleri* McLachlan, 1868, 5^th^ instar larva, head, pro- and mesothorax, right lateral view (arrow: mandibles with terminal teeth). Scale bars: 0.5 mm.

Abdomen cream colored ventrally and laterally, light purple dorsally, with chloride epithelia on abdominal segments II–VII. Abdominal segment I with 1 dorsal and 2 lateral fleshy protuberances (Fig. 4, lp, dp). Continuous transverse row of setae (some with small basal sclerites) present anterior of dorsal protuberance, comprising fused sa1, sa2, sa3 (Fig. 4), meeting dorsal sections of lateral protuberances. No setae posterior of dorsal protuberance (Fig. 4). Lateral protuberance without posterior sclerite; anterior of each lateral protuberance, a continuous band of anterolateral setae connects to dorsal and ventral sa3 setal groups (Fig. 10, bracket). Abdominal sternum I with basal sclerites of central sa2 setae fused, thereby creating multilobed pattern (Fig. 11). In total, 91–105 setae present on abdominal sterum I (Fig. 11). Abdominal segments II–VIII with 2 dorsal setae (Fig. 12, ds); only 1 posterolateral seta present on each half of abdominal dorsum IX (Fig. 12, pls). Abdominal dorsum IX with dark brown, semicircular sclerite bearing 8 long and several short setae (Fig. 13). Brown to yellow anal prolegs of limnephilid type (Fig. 12), lateral sclerite with 10 dark dorsal and row of 5 dark ventral setae, 3 of the latter very strong and prominent; ventral sole plate with black dorsal stripe and single anterior seta. Anal claw orange, with 1 small dorsal accessory hook (Fig. 14). All gills as single filaments. Dorsal gills present at most from abdominal segments II (presegmental) to VI (postsegmental positions). Ventral gills present from segments II (presegmental) to VII (postsegmental). In lateral row, gills present on segments II-III only (presegemental). Lateral fringe extends from anterior border of segment III to anterior third of segment VIII (Fig. 12, lf).

**Figures 18–23.**
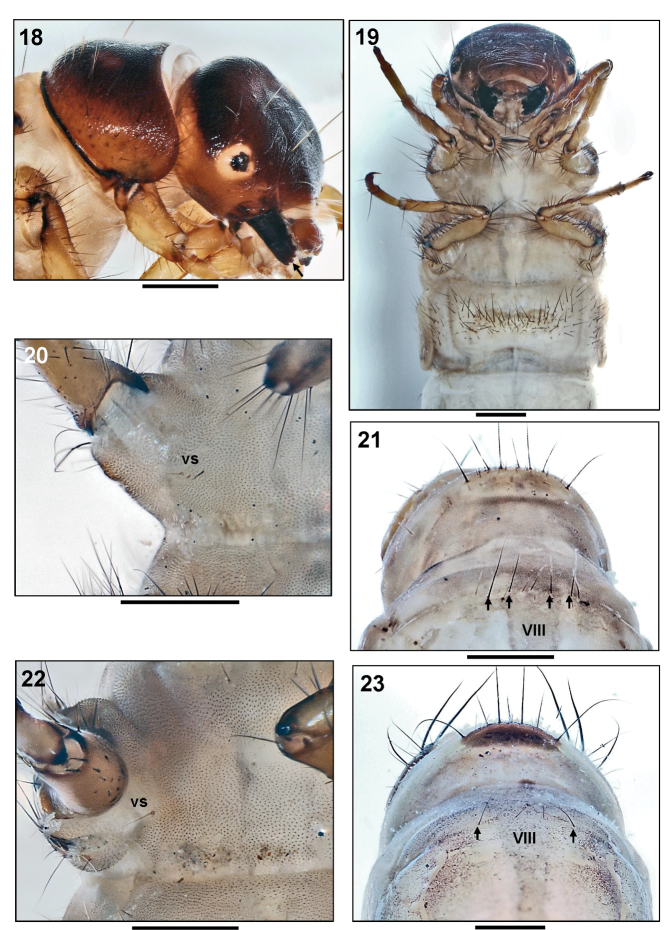
**18–21.***Drusus alpinus* Meyer-Duer, 1875, 5^th^ instar larva. **18** Head and prothorax, right lateral view (arrow: mandibles with terminal teeth) **19** Head, thorax and abdominal segment I, ventral view **20** Detail of mesosternum, ventral view (vs: ventral setae) **21** Tip of abdomen, dorsal view (arrows: long posterodorsal setae). **22–23.***Drusus franzi* Schmid, 1955, 5^th^ instar larva. **22** Detail of mesosternum, ventral view (vs: ventral seta) **23** Tip of abdomen, dorsal view (arrows: long posterodorsal setae). Scale bars: 0.5 mm.

**Figures 24–29.**
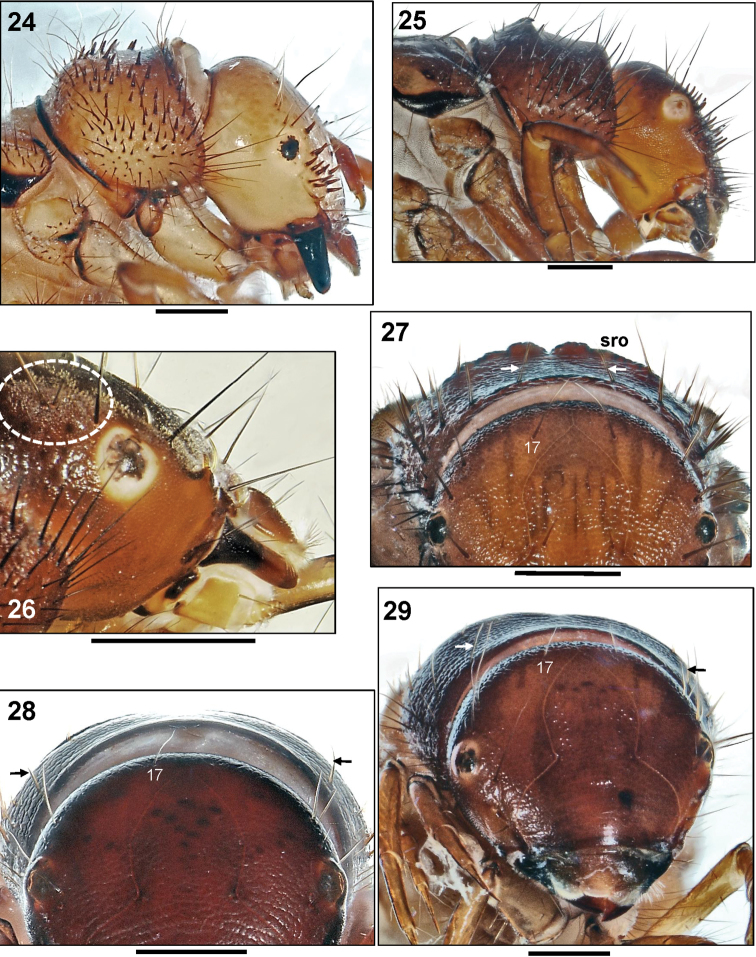
**24–25** Heads and pronota of 5^th^ instar larvae, right lateral views. **24***Ecclisopteryx dalecarlica* Kolenati, 1848 **25***Drusus botosaneanui* Kumanski, 1968 **26***Drusus serbicus* Marinkovic-Gospodnetic, 1971, 5^th^ instar larva, head, right lateral view (dotted oval: spinule area). **27–29** Heads and pronota of 5^th^ instar larvae, frontal views. **27***Drusus nigrescens* Meyer-Duer, 1875 (arrows: long dark setae reaching dorsal midline; sro: serrated outline of pronotal ridge; numbers refer to setal positions) **28***Drusus mixtus* (Pictet, 1834) (arrows: long dark setae not reaching dorsal midline; numbers refer to setal positions) **29***Drusus aprutiensis* Moretti, 1981 (arrows: long dark setae not reaching dorsal midline; numbers refer to setal positions). Scale bars: 0.5 mm.

**Figures 30–37.**
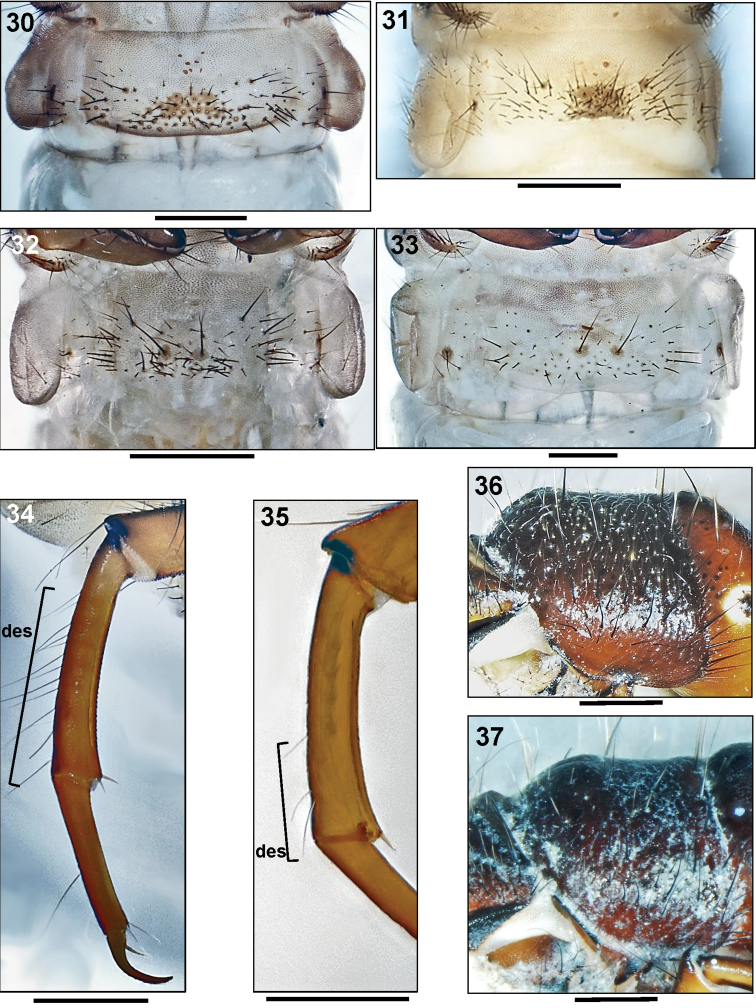
**30–33** Abdominal sterna I of 5^th^ instar larvae, ventral views. **30***Drusus nigrescens* Meyer-Duer, 1875 **31***Drusus franzressli* Malicky, 1974 **32***Drusus aprutiensis* Moretti, 1981 **33***Drusus mixtus* (Pictet, 1834). **34–35** Right hind tibiae of 5^th^ instar larvae, anterior views. **34***Drusus melanchaetes* McLachlan, 1876 (des: dorsal edge setae) **35***Drusus monticola* McLachlan, 1876 (des: dorsal edge setae). **36–37** Pronota of 5^th^ instar larvae, right lateral views. **36***Drusus vinconi* Sipahiler, 1992 **37***Drusus ingridae* Sipahiler, 1993. Scale bars: 0.5 mm.

**Figures 38–43.**
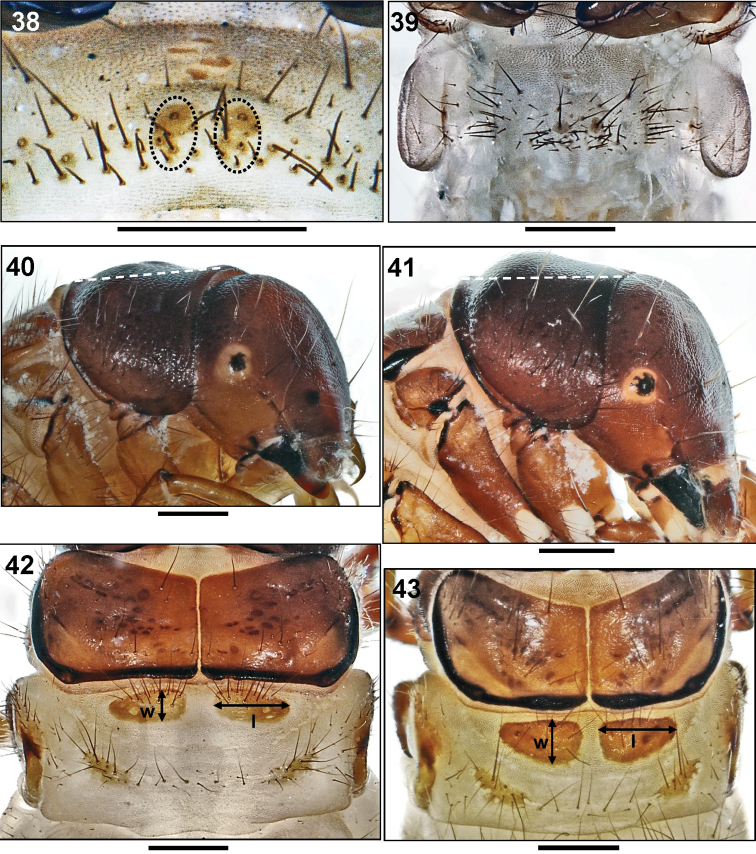
**38–39.** Abdominal sterna I of 5^th^ instar larvae, ventral views. **38***Drusus camerinus* Moretti, 1981 (dotted ovals: fused setal bases) **39***Drusus aprutiensis* Moretti, 1981. **40–41.** Heads and pronota of 5^th^ instar larvae, right lateral views. **40***Drusus aprutiensis* Moretti, 1981 (dotted line: base of flat pronotal hump) **41***Drusus croaticus* Marinkovic-Gospodnetic, 1971 (dotted line: base of high pronotal hump). **42–43.** Meso- and metanota of 5^th^ instar larvae, dorsal views. **42***Drusus croaticus* Marinkovic-Gospodnetic, 1971 (w: width, l: length of anterior metanotal sclerite) **43***Drusus mixtus* (Pictet, 1834) (w: width, l: length of anterior metanotal sclerite). Scale bars: 0.5 mm.

**Figures 44–49.**
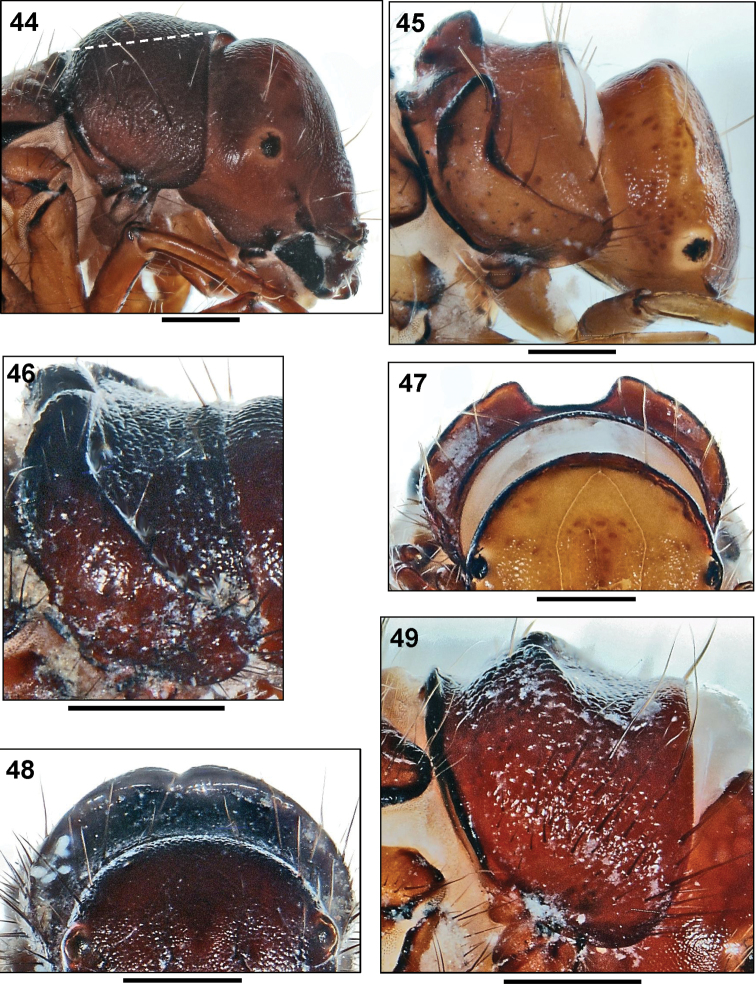
**44–46.** Heads and pronota of 5^th^ instar larvae, right lateral views. **44***Drusus mixtus* (Pictet, 1834) (dotted line: base of high pronotal hump) **45***Drusus bolivari* (McLachlan, 1880) **46***Drusus chauvinianus* (Stein, 1874) (= *Anomalopterygella chauviniana* (Stein, 1874). **47–48.** Heads and pronota of 5^th^ instar larvae, frontal views. **47***Drusus bolivari* (McLachlan, 1880) **48***Drusus chauvinianus* (Stein, 1874) (= *Anomalopterygella chauviniana* (Stein, 1874). **49***Drusus ramae* Marinkovic-Gospodnetic, 1971, 5^th^ instar larva, pronotum, right lateral view. Scale bars: 0.5 mm.

**Figures 50–57.**
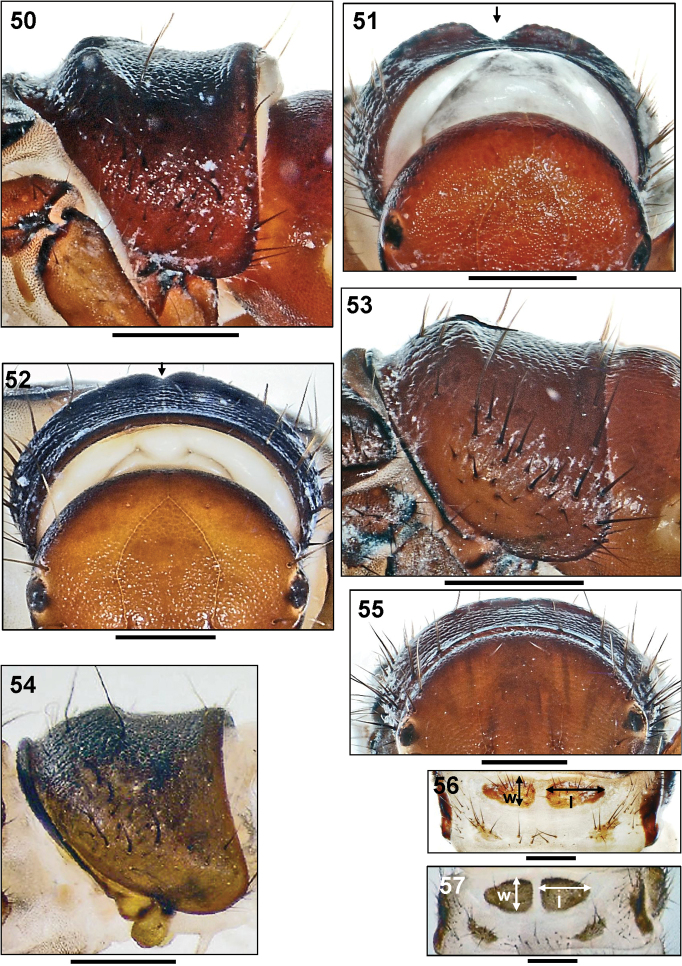
**50.***Drusus monticola* McLachlan, 1876, 5^th^ instar larva, pronotum, right lateral view. **51–52.** Heads and pronota of 5^th^ instar larvae, frontal views. **51***Drusus ramae* Marinkovic-Gospodnetic, 1971 (arrow: deep V-shaped pronotal notch) **52***Drusus monticola* McLachlan, 1876 (arrow: shallow V-shaped pronotal notch). **53–54.** Heads and pronota of 5^th^ instar larvae, right lateral views. **53***Ecclisopteryx asterix* Malicky, 1979 **54***Drusus zivici* Kučinić, Previšić, Stojanović & Vitecek, 2017. **55***Ecclisopteryx asterix* Malicky, 1979, 5^th^ instar larva, head and pronotum, frontal view. **56–57.** Metanota of 5^th^ instar larvae, dorsal views. **56***Ecclisopteryx asterix* Malicky, 1979 (w: width, l: length of anterior metanotal sclerite) **57***Drusus zivici* Kučinić, Previšić, Stojanović & Vitecek, 2017 (w: width, l: length of anterior metanotal sclerite). Scale bars: 0.5 mm.

**Figures 58–63.**
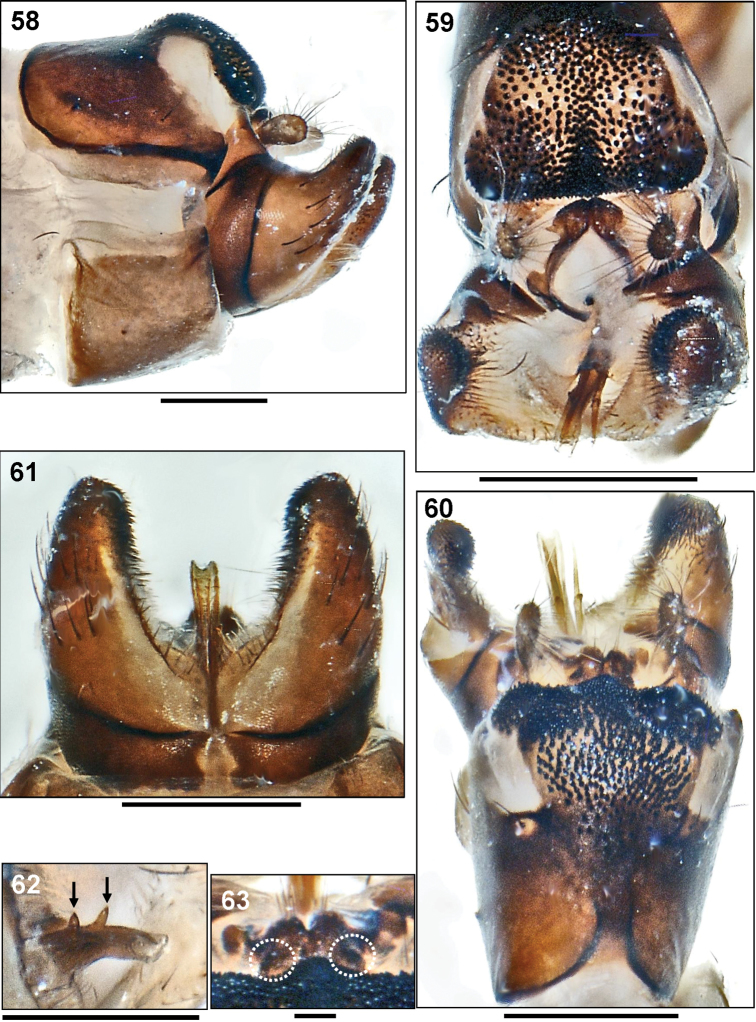
*Drusus dudor* Oláh, 2017, male genitalia. **58** Tip of abdomen, left lateral view **59** Tip of abdomen, posterior view **60** Tip of abdomen, dorsal view **61** Tip of abdomen, ventral view **62** Apex of aedeagus and parameres (arrow: dorsal tooth of parameres) **63** Detail of paraproct, dorsal view (dotted ovals: basolateral bulges). Scale bars: 0.5 mm (except Fig. 63: 0.1 mm).

#### Case.

Length 9.5–12.3 mm (*N* = 6); curved, conical (width at anterior opening 2.9–3.4 mm, at posterior opening 1.6–2.5 mm), consisting of mineral particles (sand grains and mica flakes of mixed size; Fig. 15).

#### Material examined.

ITALY: Piemonte: near Fondo, 45°30' N, 07°42' E, 1584 m a.s.l., 11 June 2019, S. Vitecek, J. Martini, 1 final instar larva, several adult males; Piemonte: Pian della Battaglia, 45°33' N, 07°39' E, 1403 m a.s.l., 25 July 2019, J. Martini, N. Falk, 4 final instar larvae; Piemonte: Lago del Gias del Prete, 45°31' N, 07°38' E, 2222 m a.s.l., 24 July 2019, J. Martini, N. Falk, 1 final instar larva, 1 male.

## Morphological separation of 5^th^ instar larvae of *Drusus dudor* from other European Trichoptera

Within the framework of the larval key by Waringer and Graf (2013), larvae of subfamily Drusinae are separated from other Trichoptera species by the following characters:

sclerites present on pro-, meso- and metanota; mesontum completely covered by 2 sclerites in close contact separated by a straight suture; metanotum incompletely sclerotized by 6 sclerites (Fig. 4);

prosternal horn present (Fig. 6, ph);

fleshy protuberances at abdominal segment I present dorsally and ventrally (Fig. 4, dp, lp);

gills consisting of single filaments only;

transverse groove typical for other limnephilids with single filament-gills (tribes Chaetopterygini, Stenophylacini) lacking at the anterior third of the pronotum (Fig. 3) except in *Drusus budtzi* (Ulmer, 1913) (= *Leptodrusus budtzi* (Ulmer, 1913) (endemic species on Corsica, Sardinia, and Mallorca and the only Drusinae species on those islands (Neu et al. 2018); larval key for Sardinia provided by Waringer and Malicky (2018).

Within the framework of Key 1 presented below (Master key for main groups of larval Drusinae), *Drusus dudor* belongs to Group C of the Drusinae grazer clade. Information for the identification of the five species belonging to Group C is given in Key 2 in the present paper.

### Key 1: Master key for main groups of larval Drusinae (final instars)

**Table:** 

1	Mandibles with terminal teeth along edges (Figs 17, 18, arrows)	**2**
–	Mandibles lacking terminal teeth along edges (Fig. 3m): **Drusinae grazer clade**	**4**
2	With filtering bristles on legs (Fig. 16, dotted oval) and abdominal sternum I (Fig. 16 fb); head capsules strongly modified (Figs 16, 17)	**Drusinae filtering carnivore clade** (Vitecek et al. 2015, Supplementary data 3; 10 species included)
–	Without filtering bristles on legs and abdominal sternum I (Fig. 19); head capsules unmodified (Fig. 19): **Drusinae shredder clade**	**3**
3	With group of 3 to 4 ventral setae close to median insertion of meso- and metacoxa (Fig. 20, vs); dorsum of abdominal segment VIII with 4 to 8 long posterodorsal setae (Fig. 21, arrows); western Alps	***Drusus alpinus* (Meyer-Dür, 1875)**
–	With only one ventral seta close to median insertion of meso- and metacoxa (Fig. 22, vs); dorsum of abdominal segment VIII with 2 long posterodorsal setae (Fig. 23, arrows); eastern Alps	***Drusus franzi* Schmid, 1956**
4	In addition to standard set of 18 pairs of primary setae, head capsule with additional spines and/or bristles (Figs 24, 25)	**Drusinae grazer clade Group A** (Waringer et al. 2015, Table 6; 10 species included)
–	Head capsule only with standard set of 18 pairs of primary setae (Figs 1, 2)	**5**
5	With spinule area posterior of each eye, surrounding bases of setae 15 and 16 (diameter of area 0.13–0.18 mm; length of spinules < 0.03 mm; Fig. 26)	**Drusinae grazer clade Group B** (Kučinić et al. 2016; Waringer et al. 2016, 11 species included)
–	Head capsule without spinule area (Fig. 3)	**6**
6	Long anterior-row setae present near pronotal midline (Fig. 27, arrows)	**7**
–	Without long anterior-row setae near pronotal midline (Figs 5, 28, 29)	**Drusinae grazer clade Group C: Key 2 of the present paper** (5 species included)
7	Basal sclerites of setae on abdominal sternum I fused to sclerotized plates or arranged in multilobed patterns (Figs 30, 31)	**Drusinae grazer clade Group D** (Waringer et al. 2015, Table 5; 9 species included)^1^
–	Basal sclerites of setae on abdominal sternum I isolated (Fig. 32, 33)	**8**
8	Dorsal abdominal gills present	**9**
–	Without dorsal abdominal gills	***Drusus carpathicus* Dziedzielewicz, 1911**
9	On mid- and hind tibia, dorsal edge setae spread over entire length of segment (Fig. 34, des)	**Drusinae grazer clade Group E**: (Waringer et al. 2008; 2 species included)
–	On mid- and hind tibia, dorsal edge setae restricted to distal third of segment (Fig. 35, des)	**10**
10	Pronotum with dorsal ridge, not evenly rounded (Figs 45, 49)	**Drusinae grazer clade Group F: Key 3 of the present paper** (6 species included)
–	Pronotum evenly rounded (Figs 36, 37)	**Drusinae grazer clade Group G**: (Waringer et al. 2013; 6 species included)

### Key 2 to final instar larvae of Drusinae grazer clade Group C

**Table:** 

1	With pronotal ridge (Fig. 3)	***Drusus dudor* Oláh, 2017** (Italy: Piemonte)
–	Without pronotal ridge (Figs 40, 41)	**2**
2	Setal bases on first abdominal sternum fused (Fig. 38)	***Drusus camerinus* Moretti, 1981** (Italy: Umbria, Marche, Lazio)
–	Setal bases on first abdominal sternum never fused (Fig. 39)	**3**
3	Pronotal hump very flat (Fig. 40)	***Drusus aprutiensis* Moretti, 1981** (Italy: Lazio, Abruzzo)
–	Pronotal hump distinct, higher (Fig. 41)	**4**
4	Length-width ratio of anteromedian metanotal sclerites ≥ 2.0 (Fig. 42)	***Drusus croaticus* Marinkovic-Gospodnetic, 1971** (Croatia, Slovenia)
–	Length-width ratio of anteromedian metanotal sclerites < 2.0 (Fig. 43)	***Drusus mixtus* (Pictet, 1834)** (Fig. 44; France, Switzerland)

### Key 3 to final instar larvae of Drusinae grazer clade Group F

**Table:** 

1	Pronotal ridge extended to anteroventral edge of pronotum (Figs 45, 46)	**2**
–	Pronotal ridge restricted to dorsal section of pronotum (Figs 49, 53)	**3**
2	In anterior view, right and left pronotal ridge separated by central, rectangular gap (Fig. 47)	***Drusus bolivari* (McLachlan, 1880)** (Iberian Peninsula)
–	In anterior view, right and left pronotal ridge meeting at center (Fig. 48)	***Drusus chauvinianus* (Stein, 1874)** (= *Anomalopterygella chauviniana* (Stein, 1874) (widespread)
3	Pronotal ridge distinct (Figs 49, 50), in anterior view with central V-shaped gap (Figs 51, 52)	**4**
–	Pronotal ridge low (Figs 53, 54), in anterior view without central V-shaped gap (Fig. 55)	**5**
4	In anterior view, central V-shaped gap deep (Fig. 51)	***Drusus ramae* Marinkovic-Gospodnetic, 1971** (Bosnia & Herzegovina)
–	In anterior view, central V-shaped gap shallow (Fig. 52)	***Drusus monticola* McLachlan, 1876** (widespread)
5	Length-width ratio of anteromedian metanotal sclerites > 1.9 (Fig. 56)	***Ecclisopteryx asterix* Malicky, 1979** (Austria, Italy, Slovenia)
–	Length-width ratio of anteromedian metanotal sclerites < 1.9 (Fig. 57)	***Drusus zivici* Kučinić, Previšić, Stojanović & Vitecek, 2017** (Serbia)

## Discussion

In their revision of subfamily Drusinae, Oláh et al. (2017) reduced the number of Drusinae genera from 8 to 2: the generic status of the monotypic genera *Anomalopterygella* Fischer, 1966, *Cryptothrix* McLachlan, 1867, *Hadimina* Sipahiler, 2002, *Leptodrusus* Schmid, 1955 and *Monocentra* Rambur, 1842 was downgraded and synonymised with *Drusus* Stephens, 1835. The same procedure was performed with the original five species included in *Metanoea* McLachlan, 1880, leaving only two Drusinae genera, *Drusus* and *Ecclisopteryx* Kolenati, 1848. In the framework of this revision based on male genital morphology, taxa were organized into ‘species groups’ and ‘species complexes’, and former species split into ‘sibling groups’. Within this framework, *D. dudor* (Figs 58–63) is considered a sibling of *D. apuanensis* Oláh, 2017, *D. lepidopterus* (Rambur, 1842), *D. liguriensis* Oláh, 2017, *D. piemontensis* Oláh, 2017, and *D. savoiensis* Coppa & Oláh, 2017 (Oláh et al. 2017). *Drusus dudor* is separated from these siblings by a pair of basolateral humps on the paraproct (Fig. 63, dotted ovals) and a single dorsal, sharply pointed spur on each paramere which lacks secondary spines on its base (Fig. 62).

*Drusus dudor* is restricted to the Italian Province of Piemonte (Oláh et al. 2017). The species was collected in small, stony streams near Fondo (Piemonte, Italy) at elevations ranging from 1403 to 2222 m above sea level. The rheophilic larva inhabits springs and spring brooks where it can be observed on the surface of boulders and large stones. According to its mouthpart anatomy, *D. dudor* is a grazer, feeding exclusively on epilithic algae and biofilm. The 36 adults of the *Drusus lepidopterus* sibling group (= *Monocentra lepidoptera* Rambur, 1842) included in the ZOBODAT database (ZOBODAT 2019) were sampled between 9 March and 6 November.

## Supplementary Material

XML Treatment for Drusus dudor

## Footnotes

In 1 of 6 specimens of Drusus dudor, a long, anterior-row seta was present near the pronotal midline which, together with the fused setal bases on abdominal sternum I, keys this species also to the Drusinae grazer clade Group D. Within this group, there is only one species (Drusus nigrescens Meyer-Duer, 1875) with a V-shaped incision in the center of a high pronotal ridge as observed in D. dudor. Both species can be easily separated by the smooth outline of the pronotal ridge in D. dudor (Fig. 5, smo), whereas this outline is serrate in D. nigrescens (Fig. 27, sro).
